# *Sigesbeckia orientalis* L. Extract Alleviated the Collagen Type II–Induced Arthritis Through Inhibiting Multi-Target–Mediated Synovial Hyperplasia and Inflammation

**DOI:** 10.3389/fphar.2020.547913

**Published:** 2020-08-28

**Authors:** Ke-Gang Linghu, Shi Hang Xiong, Guan Ding Zhao, Tian Zhang, Wei Xiong, Mingming Zhao, Xiang-Chun Shen, Wei Xu, Zhaoxiang Bian, Yitao Wang, Hua Yu

**Affiliations:** ^1^Institute of Chinese Medical Sciences, State Key Laboratory of Quality Research in Chinese Medicine, University of Macau, Macao, China; ^2^The Department of Pharmacology of Materia Medica, School of Pharmaceutical Sciences, Guizhou Medical University, Guiyang, China; ^3^College of Pharmacy, Fujian University of Traditional Chinese Medicine, Fuzhou, China; ^4^School of Chinese Medicine, Hong Kong Baptist University, Kowloon Tong, China; ^5^HKBU Shenzhen Research Center, Shenzhen, China

**Keywords:** rheumatoid arthritis, synovial hyperplasia, inflammation, *Sigesbeckia orientalis* L., SW982, MH7A

## Abstract

Excessive proliferation and inflammation of synovial fibroblasts accelerate and decorate the pathological process of rheumatoid arthritis (RA). *Sigesbeckia orientalis* L. (SO) is one of the main plant sources for Sigesbeckiae Herba (SH) which has been used traditionally in treating various forms of arthritis and rheumatic pain. However, the anti-arthritic mechanisms of SO are still not clearly understood. In this study, we investigated the therapeutic effects and the underlying mechanisms of SO against collagen type II (C II)-induced RA in rats as well as the interleukin (IL)-1β–induced human synovial SW982 and MH7A cells. For the *in vivo* studies, thirty-six Wistar male rats were randomly arranged to six groups based on the body weight, and then C II-induced to RA model for 15 days, followed by treatment with the 50% ethanolic extract of SO (SOE, 0.16, 0.78, and 1.56 g/kg) for 35 days. The results suggested that SOE significantly inhibited the formation of pannus (synovial hyperplasia to the articular cavity) and attenuated the cartilage damaging and bone erosion in the CIA-induced rats’ hind paw joints. Moreover, SOE decreased the production of C-reactive protein (CRP) in the serum and the expression of IL-6 and IL-1β in the joint muscles, as well as recovered the decreased regulatory T lymphocytes. The results obtained from the *in vitro* studies showed that SOE (50, 100, and 200 µg/ml) not only inhibited the proliferation, migration, and invasion of human synovial SW982 cells but also decreased the IL-1β–induced expression of IL-6 and IL-8 both in SW982 and MH7A cells. Besides, SOE reduced the expression of COX-2, NLRP3, and MMP9, and increased the expression of MMP2 in the IL-1β–induced SW982 cells. Furthermore, SOE blocked the activation of NF-κB and reduced the phosphorylation of MAPKs and the expression of AP-1. In conclusion, SOE attenuated the C II-induced RA through inhibiting of MAPKs/NF-κB/AP-1–mediated synovial hyperplasia and inflammation.

**Graphical Abstract:**
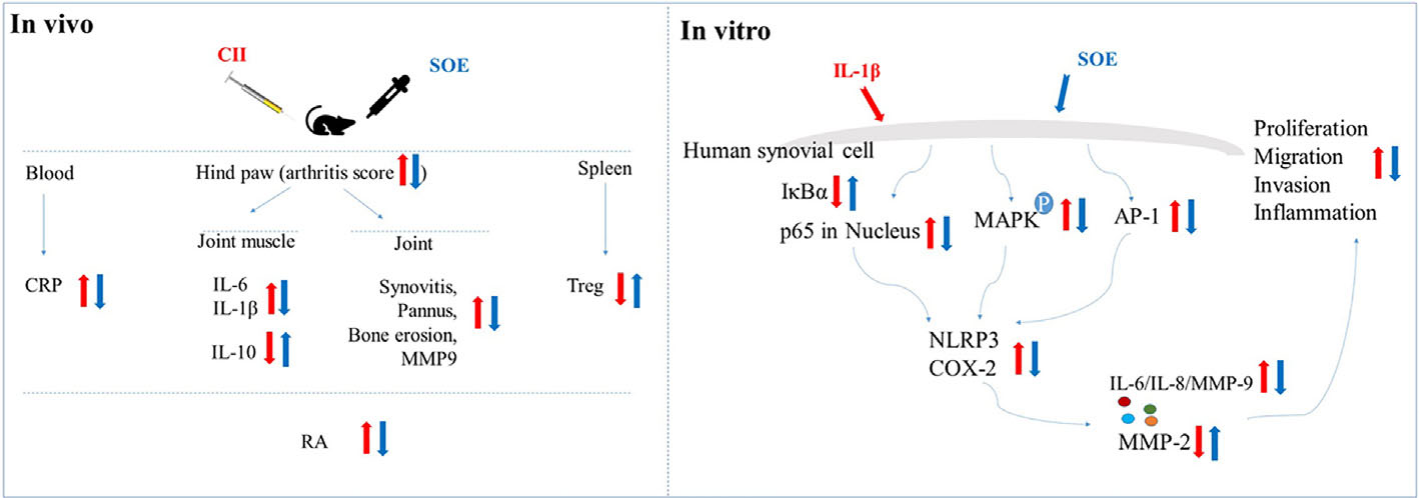


## Introduction

Rheumatoid arthritis (RA) is a common autoimmune disease characterized by immune dysfunction, chronic inflammation, synovial tissue hyperplasia, cartilage/bone damaging, and accompanied with progressive loss of joint function (Scott et al., 2010), which significantly affects the life quality of the patients and brings huge economic burden as well as the serious social problems (Scott et al., 2010). Compared with the current modern drugs (e.g. the non-steroidal anti-inflammatory agents, NSAIDs; corticosteroids; disease modifying anti-rheumatic drugs, DMARDs; and biologics, etc.) in clinical application for RA, traditional herbal medicines with the advantages of low-price, good safety, and wide cultural recognition still occupy a huge market. Investigations into the multi-biological mechanisms and quality control of herb medicines could promote the precise applications in clinic and be beneficial to such a group of people.

Sigesbeckia herba (SH) widely distributed throughout the world (Pradhan et al., 2018) is a traditional herb medicine with a long history for management of various inflammation-related diseases (especially for rheumatism) in China (Zhong et al., 2019) and some other Asian countries (Jang et al., 2018). In United States, SH was approved to be used for promoting healthy joints in dietary supplements. In 2015, a preparation with SH extract (Phynova Joint and Muscle Relief Tablets™) was licensed by Medicines and Healthcare Products Regulatory Agency (MHRA) in United Kingdom as a traditional herbal medicine for joint and muscle pain. With the increasing concerns on this herb medicine for its anti-inflammation and joint protection, it is critical to demonstrate the anti-arthritic effects and mechanisms of SH with modern research approaches.

In China, the officially authorized plant origins for SH include *S. pubescens* Makino (SP), *Sigesbeckia orientalis* L. (SO), and *S. glabrescens* Makino (SG). Huh et al. had reported that the therapeutic effect of SP on cartilage protection in a rabbit collagen type II (C II)-induced model of osteoarthritis (Huh et al., 2008). In terms of SG and SO, there is not any direct and scientific demonstration related to the RA. Hong et al. reported that SO attenuated λ-carrageenan–induced paw edema and LPS-induced systemic inflammation in mice (Hong et al., 2014), which indicated the potential of SO on RA and valuable to be further investigated. Our previous works have made a systemic analysis on the chemistry for SH species (Tao et al., 2018; Tao et al., 2020) and identified some potential quality control markers for SO (Linghu et al., 2020b). Growing *in vitro* researches showing the anti-inflammatory effects of SO on macrophages (Hong et al., 2014; Guo et al., 2018; Zhang et al., 2019; Linghu et al., 2020b), which indicates that anti-inflammation could be one of the anti-arthritic mechanisms of SO. An additional therapeutic and mechanical demonstration of SO on RA animal model and other potential target cells are essential to elucidate the anti-arthritic effects and mechanisms of SO comprehensively.

The pathophysiological mechanism of RA is very complicated. Generally, systemic immune imbalance accompanying with local inflammatory infiltration to synovium and joints go through the whole process of RA (Smolen et al., 2016). Various cells such as macrophages, lymphocytes, and fibroblasts have been identified to be participated in the development and deterioration of RA (Branimir Anić, 2014). To be an important part in synovial tissue, the synovial fibroblasts (also known as fibroblast-like synovial cells, FLS) has been demonstrated to play a key role in the destruction of joints by secreting various cytokines, proteases, and arachidonic acid metabolites (Huber et al., 2006). The activated FLS promotes the clinical symptoms and processes of RA (Ospelt et al., 2004). The excessive proliferation and invasion of FLS has been reported to be closely related to the pathogenesis of RA, effective inhibiting of cytokines and proteases through blocking or attenuating the activation of FLS might be another strategy for clinical therapeutics of RA (Xu et al., 2017).

In the present study, we investigated the therapeutic effects of SO against C II-induced RA in rats, and further revealed the anti-arthritic mechanisms of SO on IL-1β–induced human fibroblast-like synovial cells (SW982 and MH7A). Our results suggested that SO effectively alleviated the C II-induced RA through inhibiting of multi-target–mediated the synovial hyperplasia and inflammation.

## Materials and Methods

### Chemicals and Reagents

3-[4, 5-Dimethyl-2-thiazolyl]-2, 5-diphenyltetrazolium bromide (MTT), lipopolysaccharides (LPS, *Escherichia coli* O111:B4), dimethyl sulfoxide (DMSO) and indomethacin (IND) were purchased from Sigma-Aldrich (St. Louis, MO, USA). FITC anti-rat CD4, PE anti-rat CD25, Alexa Fluor^®^ 647 anti-mouse/rat/human FOXP3, (Alexa Fluor^®^ 647, PE, and FITC) Mouse IgG1, κ isotype ctrl, and True-Nuclear™ Transcription Factor Buffer Set were obtained from Biolegend (San Diego, CA, USA). Fetal bovine serum (FBS), 0.25% Trypsin-EDTA (w/v), Dulbecco’s modified Eagle’s medium (DMEM), penicillin-streptomycin (10,000 U/ml, P/S), and phosphate-buffered saline (PBS) were purchased from Thermo Fisher Scientific (Waltham, MA, USA). Interleukin (IL)-1β protein and enzyme-linked immunosorbent assay (ELISA) kits (IL-1β, IL-6, IL-10) were supplied by Neobioscience Technology Co., Ltd. (Shenzhen, China). Rat C-Reactive Protein (CRP) ELISA Kit was purchased from Life Diagnostics, Inc. (West Chester, USA). Primary antibodies against phosphorylation (P)-p65, NLRP3, COX-2, P- IκBα, IκBα, P-JNK, JNK, ERK1/2, c-Fos, c-Jun, Calreticulin (D3E6) XP^®^ Rabbit mAb (Alexa Fluor^®^ 594 Conjugate), GAPDH (glyceraldehyde-3-phosphate dehydrogenase) and the secondary antibody were purchased from Cell Signaling Technology (Danvers, MA, USA). Primary antibodies against MMP9 and MMP2 were supplied by the Abcam (Cambridge, UK). Primary antibodies against P-ERK1/2, P-p38 and p38 were obtained from Invitrogen (Carlsbad, CA, USA). Primary antibody against COX-1 was purchased from Beyotime (Shanghai, China).

### Herbs and Herbal Extracts

The herb of *sigesbeckia orientalis* L. (SO) was collected from Ganzhou (Jiangxi Province, China) and authenticated by Dr. Hua Yu (the corresponding author). The voucher specimen (No. SO-05) was deposited at the Institute of Chinese Medical Sciences, University of Macau, Macao, China. The SO extract (SOE) used in this study was prepared previously using SO material (No. SO-05). In brief, the herb was extracted twice with 50% ethanol, and the extracts were collected, filtered, concentrated, and then lyophilized. In addition, the contents of five main components (kirenol, darutoside, 3-O-methylquercetin, 16- O-acetyldarutoside, and 3,7-dimethyl-5,3′,4′-trihydroxyflavone) have been reported to be 3.69%, 2.62%, 2.38%, 3.74%, and 1.21%, respectively, by UPLC analysis (Linghu et al., 2020b).

### Experimental Animals and Treatments

Male Wistar rats (7–8 weeks old) were housed on a standard animal laboratory environment (specific-pathogen-free, regular chow diet, and water ad libitum, controlled temperature at 20°C to 22°C, relative humidity of 50% and 12-h light/dark cycle). All experimental protocols (reference number: UMARE-029-2016) complied with the National Institutes of Health guidelines for the Care of Use of Laboratory Animals and were approved by the Animal Research Ethics Committee of the University of Macau, Macau Special Administrative Region, China.

The collagen-induced arthritis (CIA) model in rats was replicated by using emulsified bovine type II collagen in Freund’s incomplete adjuvant according to manufacturing procedures (Chondrex, Inc., NE Redmond, WA 98052). Thereafter, rats were randomly divided into 6 groups (*n* = 6 in each) according to the body weight: Ctrl (vehicle), CIA (model), CIA-SOE (0.16, 0.78, and 1.56 g/kg) and CIA-indomethacin (2.5 mg/kg, positive control). The animals received saline or indicated drugs intragastrically once per day for 35 consecutive days. The body weights of the animals were monitored, and the clinical signs of arthritis were scored every week as previously described (Sims et al., 2004). At the end of the experiment, blood sample was collected from rats’ orbits, and the serum was separated for enzyme-linked immunosorbent assay. Rats were sacrificed by CO_2_ inhalation, tissues or organs were isolated on ice for indicated experiments or stored at −80°C for further analysis.

### ELISA Assay for Serum and Joint Muscles

Joint muscles were lysed by cell lysis buffer (Beyotime, Jiangsu, China), and the protein concentration was detected using a BCA protein assay kit (Thermo Fisher Scientific, MA, USA). The concentrations of serum C-reactive protein (CRP) and inflammation-related proteins (IL-6, IL-1β, and IL-10) in the lysate were determined using ELISA kit according to the manufacturer’s protocols.

### Flow Cytometry Analysis

The spleen was grinded and filtered through a 40-μm nylon cell strainer to obtain a single cell suspension. The red blood cells were then eliminated by the red blood cell lysate. Purified splenocytes (5–10 × 10^5^ cells) were surface-stained with anti-CD4-FITC/CD25-PE on ice in darkness for 30 min, then permeabilized and stained with anti-mouse/rat/human Foxp3-Alexa Fluor Stain^®^ 647 for another 30 min. The data acquisition and cell population analysis (at least 1 × 10^4^) for the splenocytes were conducted using a flow cytometer with the BD FACSDiva Software (FACSCanto, BD Biosciences).

### Radiographic and Histopathological Evaluation

On day-50, plain films of the hind paws were obtained using the IVIS Lumina XR imaging system (Clipper, Massachusetts, USA). Thereafter, the hind paw was fixed with 10% neutral phosphate buffered formalin for 7 days after removing the joint muscle. All samples were then decalcified with a mixed acid solution (containing 8% hydrochloric acid, v/v; 5% acetic acid, v/v; and 10% salicylic acid, w/v) for 2-3 weeks. The decalcified joints were paraffin sectioned for histopathological analysis by hematoxylin and eosin (H&E) (Linghu et al., 2019) and immunofluorescence staining (Chukkapalli et al., 2016).

### Culture of Human Fibroblast-Like Synoviocyte

Human synovial cells (SW982) were obtained from the American Type Culture Collection (Manassas, Virginia, USA). Rheumatoid fibroblast-like synovial cells (MH7A) were gifted by Professor Huang Runyue of Guangzhou University of Chinese Medicine. The cells were cultured in DMEM containing 10% FBS and 1% P/S at 37° C in an atmosphere of 95% humidity and 5% CO _2_. When they had grown to complete confluence, they are subcultured by trypsinization (0.25% trypsin, 0.5 mM EDTA)

### Cell Viability and ELISA Assay

Cells (1 × 10^4^ cells/well) were seeded onto 96-well plates. The cells were co-treated with the indicated concentrations of SOE for 24 h in the absence or presence of IL-1β (20 ng/ml) after adhering for 24 h. The released cytokines (IL-6 and IL-8) in the culture medium were quantitatively determined using an ELISA kit according to the manufacturer’s instructions, and the viability of the cells were detected by MTT assay (Linghu et al., 2020b).

### Scratch Wound Healing Assay

SW982 cells (1 × 10^5^ cells/well) were seeded onto a 24-well plate and allowed to form a cell monolayer. A scratch wound was built in each group with a 10-μl pipette tip and the unattached cells were discarded by PBS. The scratch wound was photographically recorded under the microscope. After co-treatment with the SOE (50, 100, 200 μg/ml) in the absence or presence of IL-1β (20 ng/ml) for 24 h, the healing of the scratch wound was comparatively assessed by the recovered wound size with the Image J software.

### Transwell Migration and Invasion Assays

The effects of SOE on the migration and invasion of SW982 cells were performed using a transwell chamber (8.0 μm pore size, Corning, USA). Cells were co-treated with the SOE (50, 100, 200 μg/ml) in the absence or presence of IL-1β (20 ng/ml) for 24 h and suspended into serum-free DMEM. The cell suspension (containing 5 × 10^4^ FLS cells) and FBS (10%)-contained DMEM (600 μl) were added into the upper and lower of transwell chamber, respectively. In parallel, similar invasion assays were conducted by adding an ECM gel (Sigma-Aldrich, USA) before adding cells to the upper chamber. After 8 h, non-migrating cells were removed with a cotton swab, then the membrane was fixed with 4% paraformaldehyde for 20 min and stained with DAPI (4′, 6-dimidyl-2-phenylxylene). Stained cells that had migrated to the undersurface of the membrane were counted in five randomly selected areas using a light microscope.

### Western Blot Analysis

SW982 cells were seeded in 6-well plates at a density of 5 × 10^5^ cells/well. Twenty-four h later, the cells were pretreated with the SOE (50, 100, 200 µg/ml) for 1 h then stimulated with IL-1β (20 ng/ml) for another 12 h. The cells were washed and collected for Western blot assay (Linghu et al., 2020a).

### Immunofluorescence Staining

SW982 cells (5 × 10^5^ cells per dish) were seeded into a confocal dish (NEST Biotechnology Co., Ltd; Jiangsu, China) and cultured for 24 h. The cells were pre-treated with SOE for 1 h and stimulated with IL-1β (20 ng/ml) for another 1 h. Subsequently, the cells were stained and analyzed as previously (Linghu et al., 2020a).

### Statistical Analysis

All data from a minimum three experiments were shown as mean ± SD. Data were analyzed on GraphPad Prism 6.0 software based on a one-way ANOVA with Dunnet’s multiple comparisons test. A value of *P*<0.05 was considered significant for all tests.

## Results

### The Effects of SOE on Animal Growth and Clinical Score of Arthritis Index

**Figure 1:**
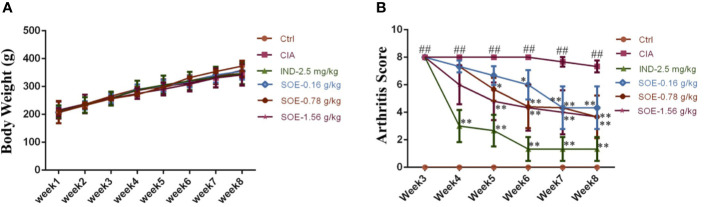
The effects of SOE on animal growth and clinical score of arthritis index. The 36 Wistar male rats were randomly divided into 6 groups according to body weight as following: Ctrl (Vehicle), CIA (collagen-induced arthritis), CIA- SOE (SO extract, 0.16, 0.78, and 1.56 g/kg) and CIA-IND (indomethacin, 2.5 mg/kg). After the CIA was reproduced, drugs were administered intragastrically daily for five weeks. The body weight was recorded every week. Clinical arthritis scores were evaluated once a week. **(A)** The growth curve of rats (*n* = 6); **(B)** The severity of arthritis by the arthritis score (^##^*P* < 0.01 vs. Ctrl group; ^*^P < 0.05 and ^**^P < 0.01 vs. CIA group, *n* = 6).

The effects of SOE on body weight and arthritic joint score of the rats were illustrated in **Figure 1**. Successful induction of arthritics (the highest score of 8 points) in 3 weeks in rats was observed after the S.C. injection of bovine type II collagen. However, the clinical signs of the arthritics could be gradually improved by oral administration of SOE or IND (**Figure 1B**). Moreover, SOE presented dose-dependent therapeutic effects in RA rats but did not influence on the animal growth during the 35 days treatment, suggesting the good safety of SOE (**Figure 1A**).

### SOE Moderated the Inflammation-Related Proteins in Serum and Joint Muscles

**Figure 2:**
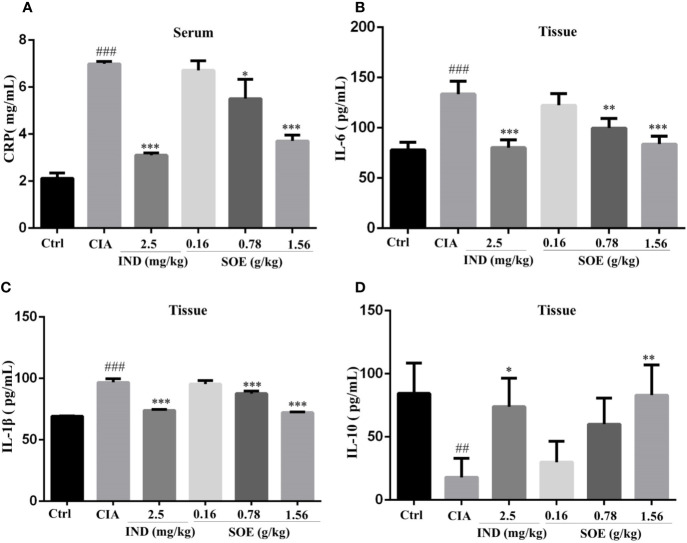
SOE moderated the inflammation-related proteins in serum and the joint muscles. On the last day, the c-reactive protein (CRP) **(A)** in the serum and IL-6 **(B)**, IL-1β **(C)** and IL-10 **(D)** in joint muscles were determined by ELISA kits. (^##^*P* < 0.01 and ^###^*P* < 0.001 vs. Ctrl group; ^*^P < 0.05, ^**^P < 0.01, and ^***^P < 0.001 vs. CIA group, *n* = 6).

As shown in **Figure 2A**, the increased expression of serum C-reactive protein (CRP) in CIA rats could be dose-dependently decreased by SOE, and with a comparable effect to that of IND. In addition, SOE also reduced the levels of pro-inflammatory proteins of IL-6 (**Figure 2B**) and IL-1β (**Figure 2C**) and promoted the anti-inflammatory protein of IL-10 (**Figure 2D**) in joint muscles. The therapeutic effects of SOE (1.56 g/kg) was comparable to that of IND (2.5 mg/kg).

### SOE Upregulated the Level of CD25^+^CD4^+^FOXP3^+^ Cells in Spleen

**Figure 3:**
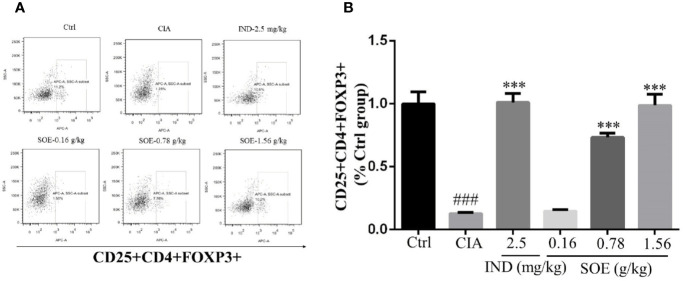
SOE upregulated the level of CD25^+^CD4^+^FOXP3^+^ cells in spleen. The purified splenocytes (5–10 × 10^5^ cells) were surface-stained with anti-rat CD4-FITC/CD25-PE for 30 min on ice in darkness, then permeabilized and stained with anti-mouse/rat/human Foxp3-Alexa Fluor^®^ 647 for another 30 min. The acquisition of flow cytometry data and the analysis of a cell population of at least 1 × 10^4^ splenocytes were performed on flow cytometer with the BD FACSDiva Software (FACSCanto, BD Biosciences). **(A)** Representative images of flow cytometry for CD4^+^CD25^+^Foxp3^+^ regulatory T cells. **(B)** Quantification of the number of CD4^+^CD25^+^Foxp3^+^ regulatory T cells. (^###^*P* < 0.001 vs. Ctrl group; ^***^P < 0.001 vs. CIA group, *n* = 6).

The fluorescence-activated cell marker analysis of CD4^+^, CD25^+^ and Foxp3^+^ cell populations in both healthy and RA rats were illustrated in **Figure 3A**. The percentages of regulatory T cells (Tregs) with CD25^+^CD4^+^FOXP3^+^ in splenocytes were detected to be 11.03 ± 0.87% (Ctr), 1.39 ± 0.09% (CIA), 11.17 ± 0.62% (CIA+IND, 2.5 mg/kg), 1.63 ± 0.11% (CIA+SOE, 0.16 g/kg), 8.09 ± 0.31% (CIA+SOE, 0.78 g/kg) and 10.90 ± 0.79% (CIA+SOE, 1.56 g/kg), respectively. By comparing the relative percentage of Tregs in each group (% of the Ctr group) (**Figure 3B**), the RA-induced low percentage of CD4^+^CD25^+^FOXP3^+^ Tregs could be up-regulated by SOE and IND effectively.

### SOE Improved Radiological and Histopathological Characteristics of Hind Paw Joints

**Figure 4:**
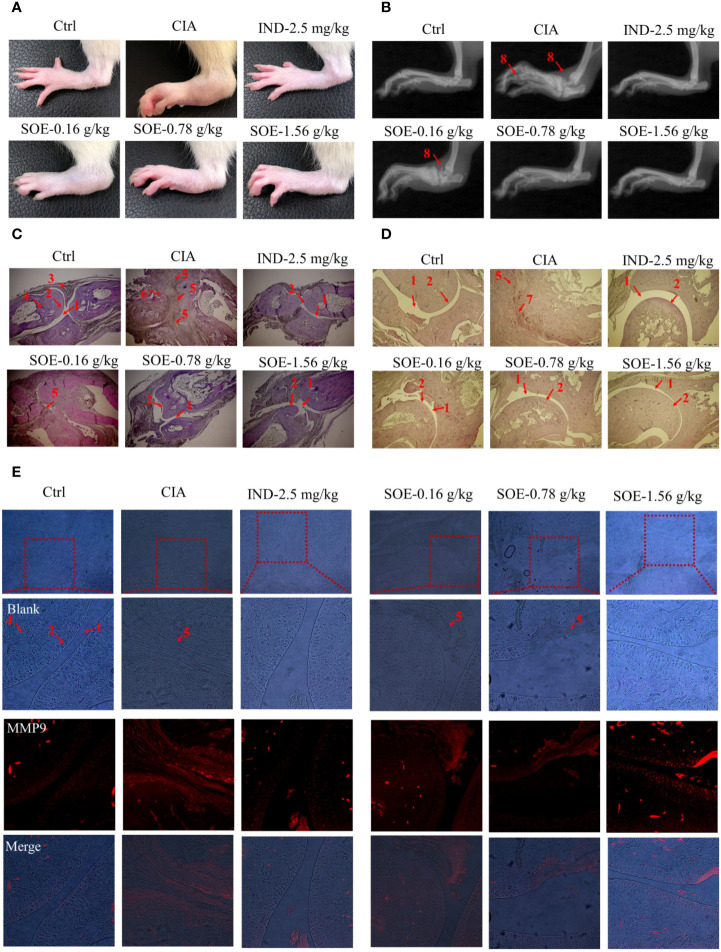
SOE improved radiological and histopathological characteristics of hind paw joints. Photographs of the hind paws **(A)**. X-ray images of the hind paws **(B)**. Histological sections of the toe joints **(C)** and ankle joints **(D)**. The expression of MMP9 in the area of pannus **(E)** (1, articular cavity; 2, cartilage; 3, synovial membrane; 4, bone; 5, pannus; 6,8, bone erosion; 7, inflammatory infiltration).

As shown in **Figures 4A, B**, oral administration of SOE or IND effectively alleviated the C II-induced hind paw swelling and joint structural damage in CIA rats. Further examined by histopathological images (**Figures 4C, D**), a complete articular cavity at the toe joint and ankle were observed in the rats of Ctrl group, while the severe synovial hyperplasia in the articular cavity (formation of pannus) accompanying with obvious expression of MMP9 (**Figure 4E**) were found at those areas in the CIA group, which finally led to the narrowed articular cavity, cartilage defect and bone erosion. However, treatment with SOE or IND significantly alleviated the abnormal proliferation of synovial cells, decreased the number of infiltrated inflammatory cells and reduced the pathological expression of MMP9, thus effectively improving the C II-induced damage of bone or cartilage.

### SOE Alleviated IL-1β–Induced Proliferation, Migration, and Invasion in the SW982 Human Synovial Cell Line

**Figure 5:**
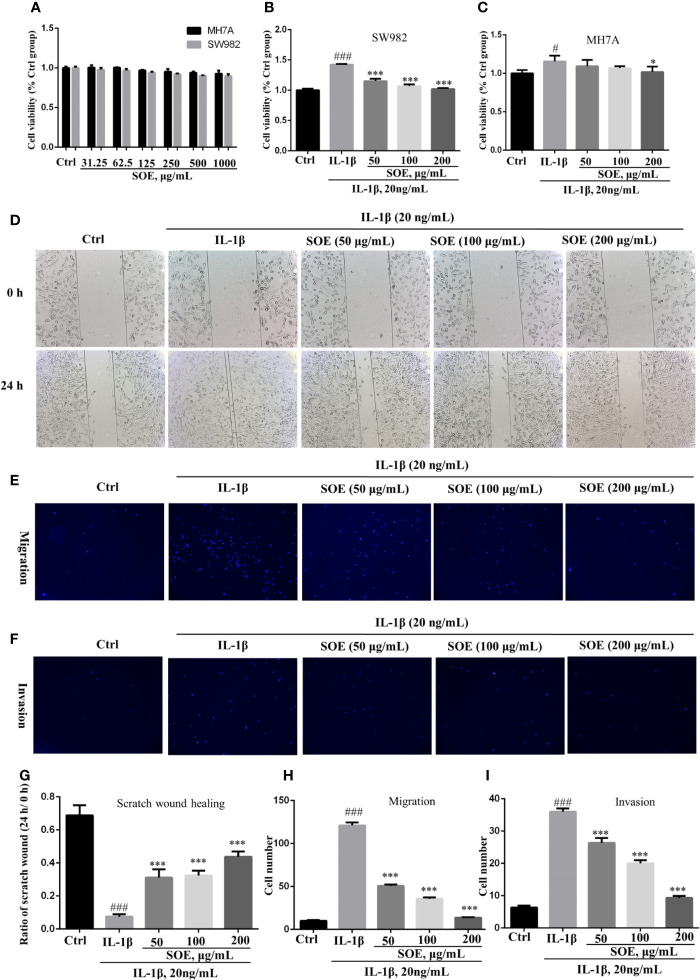
SOE alleviated IL-1β–induced proliferation, migration, and invasion in the human synovial cell line. Cells were seeded in 96-well (1 × 10^4^ cells/well), 24-well (1 × 10^5^ cells/well) and 6-wells (5 × 10^5^ cells/well) plates and allowed to grow for 24 h. A scratch wound was built in each group with a 10-μl pipette tip in the 24-well plate. Cells were co-treated with the indicated concentrations of SOE for 24 h in the absence or presence of IL-1β (20 ng/ml). The co-treated cells were transferred into transwell plates to analyze the migration and invasion. The cytotoxicity of SOE to MH7A and SW982 cells **(A)**. Inhibition of SOE on IL-1β–induced proliferation **(B, C)**, migration **(D, E)** and invasion **(F)** in the human synovial cell line. Statistical data for scratch wound healing **(G)**, migration **(H)** and invasion **(I)**. (^#^*P* < 0.05, ^###^*P* < 0.001 vs. Ctrl group; ^*^P < 0.05, ^***^P < 0.01 vs. IL-1β group, *n* = 3).

The MTT assay indicated that SOE was not obviously toxic to SW982 and MH7A cells under the concentrations less than 1,000 μg/ml in 24 h (**Figure 5A**), and presented an effective alleviation on IL-1β–induced cell proliferation in the concentrations ranged from 50 to 200 μg/ml (**Figures 5B, C**). Moreover, SOE was further observed to dose-dependently inhibit the IL-1β–induced SW982 cell migration (**Figures 5D, G, E, H**) and invasion (**Figures 5F, I**) through the scratch wound test and transwell assays.

### SOE Reduced the Production of IL-6 and IL-8 in SW982 and MH7A Cells

**Figure 6:**
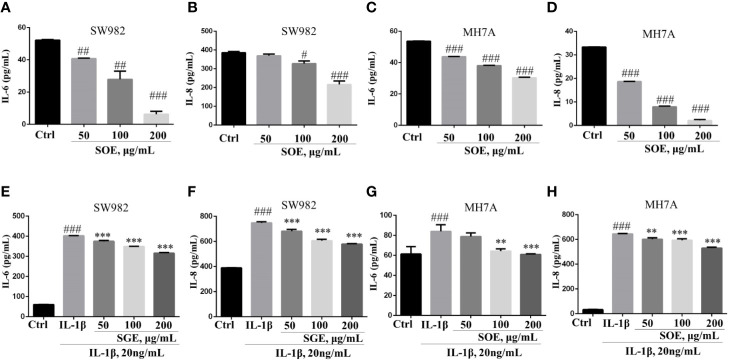
SOE reduced the production of IL-6 and IL-8 in SW982 and MH7A cells. Cells were seeded in 96-well plates (1 × 10^4^ cells/well) and allowed to grow for 24 h. Cells were co-treated with SOE (50, 100, 200 μg/ml) for 24 h in the absence or presence of IL-1β (20 ng/ml). Thereafter, the cytokines of IL-6 **(A, C, E, G)** and IL-8 **(B, D, F, H)** in the supernatant was quantified using ELISA kits according to the manufacturer’s instructions. (^#^*P* < 0.05, ^##^*P* < 0.01, ^###^*P* < 0.001 vs. Ctrl group; ^**^P < 0.01, ^***^P < 0.001 vs. IL-1β group, *n* = 3).

As shown in **Figure 6**, SOE (50, 100, and 200 μg/ml) was detected to inhibit the IL-1β–induced over-production of IL-6 and IL-8 in SW982 (**Figures 6E, F**) and MH7A (**Figures 6G, H**) cells dose-dependently. Additionally, SOE could also attenuate the basal level of IL-6 and IL-8 production in SW982 (**Figures 6A, B**) and MH7A (**Figures 6C, D**) cells when treatment with SOE for 24 h in the absence of IL-1β.

### SOE Reduced the Expression of COX-2, NLRP3, and MMP9 but Increased the Expression of MMP2 in IL-1β–Induced SW982 Cells

**Figure 7:**
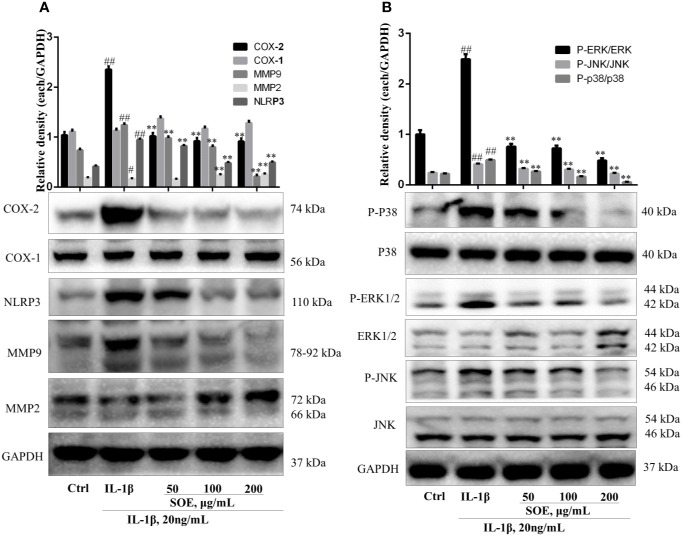
SOE reduced the expression of COX-2, NLRP3, MMP9, and the phosphorylation of MAPKs but increased the expression of MMP2 in IL-1β–induced SW982 cells. SW982 cells were seeded in 60 mm cell culture dish (1 × 10^6^ cells/well) and allowed to grow for 24 h. Cells were pretreated with SOE (50, 100, 200 μg/ml) for 1 h, then stimulated with IL-1β (20 ng/ml) for 12 h or 1 h. The expression levels of COX-1, COX-2, NLRP3, MMP9, MMP2 **(A)** and MAPKs **(B)** were detected by Western blotting. (^##^*P* < 0.01 vs. Ctrl group; ^**^P < 0.01 vs. IL-1β group, *n* = 3).

The changes of several proteins (i.e. COX-2, NLRP3, MMP9, and MMP2) expressed on the synovial tissue were associated with the initiation to the deterioration of RA. As shown in **Figure 7A**, IL-1β induction increased the expression of COX-2, NLRP3, and MMP9 but decreased the expression of MMP2 in human synovial SW982 cells, which could be reversed by the treatment of SOE in a dose-dependent manner.

### SOE Blocked the Activation of NF-κB, MAPKs, and AP-1 Signal Pathways in IL-1β–Induced SW982 Cells

**Figure 8:**
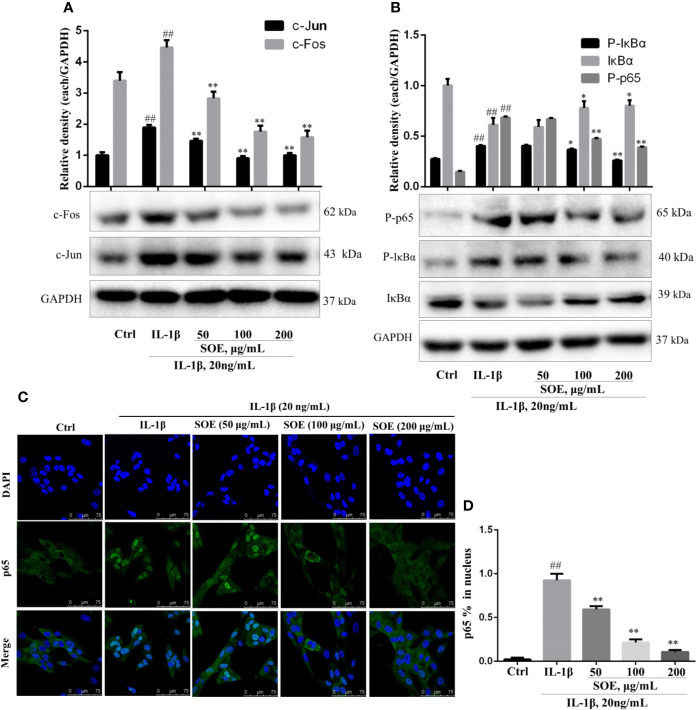
SOE blocked the activation of NF-κB and AP-1 signal pathways in IL-1β–induced SW982 cells. Cells were seeded in 60 mm cell culture dish (1 × 10^6^ cells/well) or confocal dish (5 × 10^5^ cells/well) and allowed to grow for 24 h. Cells were pretreated with SOE (50, 100, 200 μg/ml) for 1 h, then stimulated with IL-1β (20 ng/ml) for 12 h or 1 h. The expression of AP-1 **(A)** and NF-κB **(B)**, as well as the translocation of NF-κB p65 **(C, D)** were detected by Western blotting or immunofluorescence staining (^##^*P* < 0.01 vs. Ctrl group; ^*^P < 0.05 and ^**^P < 0.01 vs. IL-1β group, *n* = 3).

NF-κB is a critical signaling pathway involved in the development of RA, the phosphorylation and degradation of IκBα and the phosphorylation of NF-κB p65 (P-p65) enhanced the entrance of NF-κB p65 into the nucleus thus mediating the transcription of inflammatory genes. As illustrated in **Figure 7**, IL-1β induced the phosphorylation and degradation of IκBα but increased the expression of P-p65 (**Figure 8B**) and the translocation of p65 from cytoplasm into nucleus (**Figures 8C, D**), which could be reversed significantly by treating with SOE. MAPKs family including P38, ERK1/2, and JNK were reported to be associated with the disease process of RA through mediating the inflammation and MMPs. As shown in **Figure 7B**, IL-1β induced the phosphorylation of P38, ERK1/2, and JNK, which could be reduced by the treatment of SOE. AP-1 is another critical protein widely identified and reported in the arthritic tissue. As shown in **Figure 8A**, SOE decreased the IL-1β–induced expression of c-Jun and c-Fos.

## Discussion

Rheumatoid arthritis (RA) is a chronic disease that eventually leads to joint damage and bone erosion. The severe disabling risk of RA has brought a huge economic burden to society. With the innovation of drug development technology, RA treatments from the non-steroidal anti-inflammatory agents (NSAIDs) to biologics have obtained some achievements on improving the life quality of patients. However, due to the toxicities to liver and kidney of chemical drugs and the high price of biologics, a large number of people would prefer to choose the traditional medicines as their long-term drug treatment approaches. In such a circumstance, it is essential to conduct a systemic and scientific evaluation including the quality control, pharmacology, and pharmacokinetics on the traditional herb medicine, which could help such a group of patients to rationally use the traditional herb medicine.

*Sigesbeckia orientalis* L. (SO) is a plant source of Sigesbeckiae Herba (SH) which has been traditionally used for rheumatism in China since Tang dynasty. Because of the long history of use for this medicine, Chinese people would conventionally use the herb medicine to treat RA as a single medicine or a formula. In the past years, we have made a systemic identification on the active components and a preliminary evaluation on the pharmacokinetics. An additional therapeutic and mechanical demonstration of SH on RA animal model is beneficial to contribute the rational use of this herb medicine. Hong et al. reported that SO attenuated λ-carrageenan–induced paw edema and LPS-induced systemic inflammation in mice (Hong et al., 2014), which indicated the potential of SO on RA. At present study, we investigated the anti-arthritic effects of SO extract (SOE) on collagen type II (C II)-induced rheumatoid arthritis (CIA). As shown in **Figure 1A**, the body weight of rats between the control and SOE was no difference, which indicated the SOE was non-toxicity to rats. Moreover, SOE attenuated the RA symptom with the reduced clinical scores (**Figure 1B**).

Generally, continuous dysfunction of immune system and local inflammatory infiltration to the joints would be the main causes of RA. Production of abnormal autoantibodies usually leads to the activation of T/B lymphocytes and the interaction with macrophages to produce multiple inflammatory cytokines. These cytokines further act on the synovial membrane and cartilage, thus initiating excessive proliferation and invasion of synovial fibroblasts to the articular cavity and producing a lot of proteases and arachidonic acid metabolites, which finally leads to the joint damage and bone erosion. CD4^+^CD25^+^FOXP3^+^ T lymphocytes are the critical regulatory T cells (Tregs) to maintain the function of immune system and be reported to involve in the RA development process. Lu et al. reported that stimulation of Kirenol (one of the active compounds of SOE) on the CD4^+^CD25^+^Foxp3^+^ Tregs altered potent antiarthritic effect against CIA. Therefore, we detected the effects of SOE on the Tregs, our results show that the proportion of CD4^+^CD25^+^FOXP3^+^ T lymphocytes in the spleen of CIA rats was much lower than those of vehicle rats, which could be upregulated by treating with SOE (**Figures 3A, B**). C-reactive protein (CRP) is a ring-shaped pentameric protein existed in plasma with response to inflammation (Mortensen, 2001). The concentration of CRP has been reported to be dramatically increased in the peripheral blood in RA patients (Kowalski et al., 2008). As shown in **Figure 2A**, SOE effectively reduced the increase of CRP level in CIA rats, which might be correlated with the rebalanced immune system (**Figures 3A, B**). Moreover, SOE ameliorated the swelling of hind paw induced by C II (**Figure 4A**), which were consistent with the decreased expression of IL-6 and IL-1β and increased IL-10 in the joint muscles (**Figures 2B–D**). In addition, after treatment with SOE, the proliferation of synovial cells was alleviated, the number of infiltrated inflammatory cells and proteases were significantly decreased (**Figures 4C, D**), and the damages of bone or cartilage were also obviously improved (**Figure 4B**).

The significant inhibitory effects of SOE on synovial hyperplasia and inflammation in rats inspired us to further investigate the biological mechanisms of SOE against RA using the human fibroblast-like synoviocytes (FLS). The most commonly used cell lines on studying synovitis in RA is the human synovial sarcoma cell line (SW982) and a human rheumatoid arthritis synovial cell line (MH7A), which have been successfully used to investigate the effects of drugs and phytochemicals (Yamazaki et al., 2003; Chang et al., 2014; Khansai et al., 2017).

In consistent with the *in vivo* results, SOE reduced the expression of pro-inflammatory cytokines and chemokines on SW982 and MH7A synovial cells in the presence or absence of IL-1β induction (**Figures 5A–H**), the SOE also alleviated the IL-1β–induced proliferation, migration, and invasion in the human SW982 synovial cell line (**Figures 6B–I**). Besides, SOE did not affect the expression of COX-1 but reduced the expression of COX-2 and NLRP3 (**Figure 7A**) which were pivotal regulators of inflammation in RA. t COX-2 activity is known to be upregulated in the RA synovium (Woods et al., 2003). The selective COX-2 inhibitors such as Rofecoxib and Celecoxib have emerged as an important option in the treatment of RA (Sundy, 2001). Zhang et al. reported that extent of synovial NLRP3 expression was correlated with the clinical severity of arthritis and radiological scores (Zhang et al., 2016). SOE also reduced the expression of MMP9 while increased the expression of MMP2 in IL-1β–induced SW982 cells (**Figure 7A**), Xue et al. reported that endogenous MMP-2 or MMP-9 contribute to RA synovial fibroblast survival, proliferation, migration, and invasion, with MMP-9 having more potent effects. More importantly, MMP-9 stimulates RA synovial fibroblast-mediated inflammation and degradation of cartilage, whereas MMP-2 inhibits these parameters (Xue et al., 2014). Unlike MMP-2 KO mice, MMP-9 KO mice showed reduced levels of Antibody-Induced Arthritis, indicating that MMP-9 enhances arthritis in this model (Itoh et al., 2002). Our results revealed that SOE rebalanced the expression of MMP-2 and MMP-9 induced by IL-1β in synovial SW982 cell, this might be one of the reason that SOE inhibited the synovial inflammation and degradation of cartilage in the joint of CIA rat.

According to our previous results from computational predictions, RA was frequently involved in multiple signal pathways (Wang et al., 2019). The interactions of these pathways were associated with pathological mechanisms of RA. Based on the current reports and references, modulation on several signal pathways have been reported to be successful approaches to treat RA. Among them, the transcription factor NF-κB has been well recognized as a potential therapeutic target (Roman-Blas and Jimenez, 2006), and a pivotal regulator of inflammation, hyperplasia, and tissue destruction in rheumatoid arthritis (Makarov, 2001). Several previous studies have revealed that AP-1 (c-Fos and c-Jun) are highly expressed in the RA synovium (Kinne et al., 1995; Dooley et al., 1996). AP-1 binding sites have been identified in the promoter region of all matrix metalloproteinases (MMPs) (Benbow and Brinckerhoff, 1997), which indicates AP-1 might play a pivotal role in the transcriptional activation of MMPs. Selective inhibition of c-Fos/AP-1 resolves arthritis in a preclinical model of the disease by inhibiting the production of arthritis upstream of inflammatory cytokine and MMPs (Aikawa et al., 2008). Upstream of NF-κB and AP-1, mitogen-activated protein kinases (MAPKs) are highly active in chronic synovitis and also involved in the regulation of MMP expression (Schett et al., 2000). Three MAPK families including p38, extracellular signal-related kinase (ERK) and c-Jun N-terminal kinase (JNK) seem to be involved in the activation of synovial cells in RA (Meyer and Pap, 2005). MAPKs have been implicated in transducing inflammation and joint destruction and therefore are key molecular targets for therapeutic intervention of RA (Thalhamer et al., 2008). In previous reports, SOE have been reported to inhibit the activation of NF-κB and the phosphorylation of MAPKs on LPS-induced RAW264.7 macrophages to attenuate inflammation (Hong et al., 2014; Linghu et al., 2020b). Leocarpinolide B, an active compound from SH, significantly inhibited the NF-κB activation therefore ameliorating the inflammation and stress oxidant (Linghu et al., 2020a). Kirenol, one of the main compounds of SH, has been shown to inhibit the phosphorylation of NF-κB in cytokines-treated primary RA-synovial fibroblasts (Wu et al., 2019). Therefore, we investigated the effects of SOE on these three most important signal pathways. As shown in **Figures 8B–D**. our results showed that SOE blocked the activation of NF-κB by reducing the degradation of IκBα, the phosphorylation, and the translocation of NF-κB p65 in IL-1β–induced human synovial SW982 cells. Also, SOE decreased the expression of AP-1 (**Figure 8A**) and the phosphorylation of MAPKs (**Figure 7B**).

In summary, SO is traditional herb medicine with a long history of application. The culture recognition, low price, and safety of this herbal medicine still attracted a lot of patients and widely used in modern society. Based on the previous study on the quality control and pharmacokinetics, this present research evaluated the anti-arthritic effects and mechanisms of SOE on treating RA. Results from the *in vivo* and *in vitro* studies suggested that therapeutic effects of SOE on RA. The mechanisms of action would mainly be the rebalancing of immune system, inhibition of synovial hyperplasia and inflammation through targeting MAPKs/NF-κB/AP-1 signal pathways, suggesting the multi-targets of SO on RA treatment. Our work suggested that SOE would be an effective therapeutic drug on RA, but the doctors and patients should pay more attention on the additive effects on targets when taking a combination use of western drugs (i.e. NSAIDs, selective COX-2 inhibitors) and this herb medicine.

## Data Availability Statement

The raw data supporting the conclusions of this article will be made available by the authors, without undue reservation.

## Ethics Statement

The animal study was reviewed and approved by the Animal Research Ethics Committee of the University of Macau, Macau Special Administrative Region, China.

## Author Contributions

HY and K-GL conceived and designed the study. K-GL, SX, GZ, TZ, WXiong, and MZ conducted the experiments. X-CS, WXu, ZB, and YW provided the technical support and advices for the study. K-GL wrote the manuscript, and HY revised the manuscript. All authors contributed to the article and approved the submitted version.

## Funding

This work was supported by grants from the National Natural Science Foundation of China [NSFC, No. 81470170], the Science and Technology Development Fund of Macau SAR [File No. 0096/2019/A2], the Research Committee of the University of Macau [MYRG2017-00178-ICMS and MYRG2018-00043-ICMS], and the international Cooperation Department of National Administration of traditional Chinese Medicine [GZYYGJ2019042].

## Conflict of Interest

The authors declare that the research was conducted in the absence of any commercial or financial relationships that could be construed as a potential conflict of interest.
